# Evaluation of colour change, marginal adaptation, fracture strength, and failure type in maxillary and mandibular premolar zirconia endo-crowns

**DOI:** 10.1186/s12903-024-04755-z

**Published:** 2024-08-21

**Authors:** Mohammed Y Tarrosh, Mohammed M Al Moaleem, Aalaa Ibrahim Mughals, Raghad Houmady, Asma A. Zain, Alkhansa Moafa, Maram A. Darraj, Loay Ebrahim Najmi, Hashim A Bajawi, Shaima Abdoh Mohammed, Mohmed Isaqali Karobari

**Affiliations:** 1 Department of Restorative Dental Science, College of Dentistry, Jazan University, Jazan, 45142 Saudi Arabia; 2 Department of Prosthetic Dental Science, College of Dentistry, Jazan University, Jazan, 45142 Saudi Arabia; 3 College of Dentistry, Jazan University, Jazan, 45142 Saudi Arabia; 4 Ministry of health, Riyadh, Kingdom of Saudi Arabia; 5 Department of Dental Research, Saveetha Medical College and Hospitals, Saveetha Institute of Medical and Technical Sciences, Saveetha University, Chennai, 602105 Tamil Nadu India; 6 Department of Restorative Dentistry & Endodontics, Faculty of Dentistry, University of Puthisastra, Phnom Penh, 12211 Cambodia

**Keywords:** Endodontics, Endocrown, Zirconia, Colour measurement, Marginal adaptation, Fracture strength, Failure type

## Abstract

**Objectives:**

The objective of this in vitro study was to evaluate the effects of different preparation designs on the mean colour change (ΔE^*^), marginal adaptation, fracture resistance, and fracture types of maxillary and mandibular premolar endocrowns (ECs).

**Methodology:**

A total of 40 extracted maxillary and mandibular premolars were treated endodontically, and each type was subdivided according to the remaining axial height (remaining walls on all surfaces; 2–4 mm) and 2 mm inside the pulp chamber. Specimens were immersed in coffee for 14 days, ΔE^*^ was determined, marginal adaptation was observed, fracture forces test was conducted, and the samples were examined visually at 10× magnification to evaluate failure type and identify fracture origin. The data were entered and analyzed using Statistical Package for Social Sciences, and significance between and within groups was evaluated through ANOVA. The p-value ≤ 0.05 was considered statistically significant.

**Results:**

The ΔE* values of the maxillary premolar with 2 mm axial height were the highest (6.8 ± 0.89 units), whereas the lowest value was observed in the mandibular premolar with 4 mm axial height (2.9 ± 0.53 units). Significant differences (*p* < 0.05) in teeth and design were observed. The marginal adaptation of the mandibular premolar with 4 mm axial height was the highest (30.20 ± 1.53 μm), whereas the lowest marginal adaptation was observed in the maxillary premolar with 2 mm axial height (14.38 ± 0.99 μm), and the difference was statistically significant (*p* < 0.05). The maximum fracture force was observed in maxillary premolars with 2 mm axial height (2248.15 ± 134.74 N), and no statistically significant difference (*p* = 0.07) was observed between maxillary and mandibular premolars at 4 mm axial height.

**Conclusion:**

The recorded ΔE^*^ values of the ECs were within clinically acceptable values or slightly higher, and the marginal adaption values were within acceptable and recommended clinical values in µm. EC preparation with 2 mm axial height in both arches recorded the highest fracture forces. Type III (split fracture) failure was recorded as the highest in the maxillary and mandibular premolar ECs with different axial wall heights.

## Introduction

A premolar tooth usually requires complex restoration after root canal treatment (RCT). Post cores and crowns have several contraindications, although alternatives for restoration are available [1]. Using endocrown (EC) is an option for restoring severely damaged teeth and improving the performance of premolars. The preparation design should provide sufficient retention, stability, and structural durability for restoration [1, 2]. The EC is an adhesive restoration with minimally invasive preparation [2]. It has been widely adopted in the conservative treatment of premolar teeth owing to advances in adhesive and bonding techniques, such as monoblock restoration [3–5]. RCT restored without posts and that restored with posts have similar fracture resistance and failure modes [4].

The principles of EC preparation design have not been defined, and preparation designs previously described include cuspal reduction of 2–3 mm, 90-degree butt margins, smooth internal transition, six-degree pulp chamber taper, flat pulpal floor with sealed radicular spaces, supra-gingival enamel margins, and EC pulp chamber depth necessary for adequate retention or resistance [1, 2, 6].

The colour stability of CAD/CAM prostheses indicates the efficacy of aesthetic zone restorations and is usually measured by mean colour change (ΔE*). Coffee results in the discolouration of prostheses made primarily of zirconia and ceramic materials [7–9]. Al Ahmeri et al. [10] reported that the mean colour change in an EC CAD/CAM ceramic material was marginally higher than the acceptable values (1.7–3.9 units) [11]. Alrabeah et al. [12] found that home bleaching had the least significant effect on the ΔE* of zirconia ceramics among the tested methods. Donmez et al. [13] compared the ΔE* values of lithium disilicate, zirconia-reinforced lithium silicate, and nano-lithium disilicate before coffee thermocycling and those after coffee thermocycling and found a variance among the three materials but it was nonsignificant. The ΔE* values of a resin-based nanoceramic significantly decreased with increasing thickness (2–4 mm) to a higher degree than those of glass ceramics [14]. The immersion of zirconia ceramic materials with conventional thickness in coffee had ΔE* values within acceptable ranges [7–9].

Zirconia has been used to fabricate ECs since 2017 [15]. Zirconia CAD/CAM materials in different forms are characterized by good mechanical and bonding strength, tooth structure, and aesthetic appearance [4, 5, 16]. Zirconia with lithium disilicate glass-ceramic (LDGC) is used for EC constructions in the posterior area [17, 18]. Most general practitioners prefer zirconia as a material for EC construction in the posterior area over LDGC [18]. Monolithic zirconia eliminates persistent problems, such as bone-white opaqueness and porcelain veneer fracture. It has a high flexural strength (600–800 Mpa) [19].

Maxillary premolars are used in testing the fracture forces of EC, which are approximately 1500 N, regardless of the number of remaining walls [20]. The values range from 857 N to 1391 N when different ferrule designs are used [21]. Zirconia used for EC showed low fracture force (400 N) even with a deep extension inside the pulp with butt joint tooth preparations [22]. The values for zirconia ECs constructed in premolars are less than 1000 N [5] but higher in other studies [20, 21].

From a biomimetic perspective, preserving and conserving the tooth structure are essential for restoring the balance among the dental element’s biological, mechanical, adhesive, functional, and aesthetic factors [23]. After RCT, ECs constructed by zirconia with CAD/CAM systems have recently been used for crowning posterior maxillary and mandibular premolar teeth. These ECs have a high percentage of survivability, durability, and success rate that has been reported to be the same or more than post-and-core and crown with the advantages of conservative preparation, adhesive retention mechanism, few clinical and laboratory sessions, and the possibility of use in teeth in the presence of full or partial ferrule [4, 5, 16, 20].

Marginal adaptation is generally evaluated by measuring the marginal gap, described as the distance between a restoration’s internal surface and a preparation’s finish line [24]. McLean and von Fraunhaufer [25] concluded that 120 μm is the maximum tolerable marginal opening. Inadequate marginal adaptation can cause plaque accumulation, microleakage, caries, and endodontic inflammation, resulting in restoration failure [26]. Factors affecting the marginal integrity are the remaining enamel and dentin amount, type of impression or scanning, cementation process, bulk and type of materials, and preparation design [27, 28]. Different methods can evaluate a marginal area, including microscopy, microcomputed tomography, silicone replication, and laser videography. Direct microscopic examination of the marginal area is the most widely used method because it is non-destructive and repeatable [4, 5, 21, 26]. ECs zirconia with premolars is rarely used in measuring marginal adaptation despite its use resulting in a clinically acceptable value of marginal adaptation [4, 5].

An adequate extension of the maxillary and mandibular premolar ECs to the pulp chamber is necessary for optimal retention and resistance to EC during force application. The effects of colour changes and the marginal adaptation of maxillary and mandibular premolars have not been studied. Thus, the present in vitro study aimed to evaluate the effect of different preparation designs with short pulp chambers on the mean colour change (ΔE*) values, marginal adaptation, and fracture forces. In addition, the percentage of the failure mode of premolar ECs was assessed. The null hypothesis was that no variation in mean colour change, marginal adaptation, fracture forces, and failure type percentages would occur among EC restorations.

## Methodology

### Study design, ethical approval, and sample size calculations

This laboratory study was conducted on 40 human mandibular and maxillary premolar teeth extracted for orthodontic reasons. The teeth were collected from private dental centres in Jizan City, Jazan, Saudi Arabia. The institutional ethical review board was obtained for this study protocol and approved on January 30, 2023, with registry number REC-44/07/501. The sample size was calculated using G*Power software (version 3.1; University of Dusseldorf). The effect size (d) was 0.5, α was 0.05, and 1-β (power) was 0.65. The sample size obtained was 40 specimens, which was calculated according to the fracture resistance of the conventional EC from earlier studies [10, 29, 30].

### Inclusion criteria

Inclusion criteria for the extracted premolar teeth were nearly identical crown and root dimensions, sound teeth, and carious free from restoration, cracks, or previous RCT. The occluso-cervical height, mesiodistal and buccolingual width, premolar root length, canal morphology, and cementoenamel junction (CEJ) dimensions were nearly the same, and the mean dimensions were as follows: buccolingual, 8.72 ± 0.5 mm; mesiodistal, 9.13 ± 0.5 mm; clinical crown height from the tip of crown to CEJ, 8.5 ± 0.5 mm; and root length, 14.00 ± 0.5 mm. These dimensions were confirmed clinically by using intraoral radiographs. The sound teeth extracted for orthodontic reasons are collected and stored in 10% formalin immediately after extraction [31].

### Grouping and tooth preparations

**Fig. 1:**
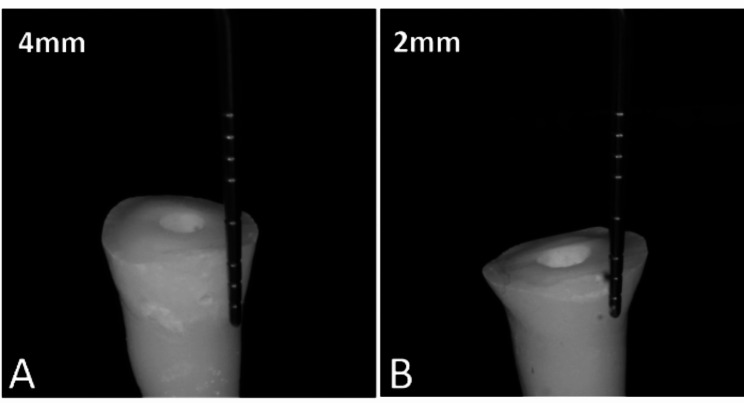
Teeth with 4 mm (**A**) and 2-mm (**B**) axial height

**Fig. 2:**
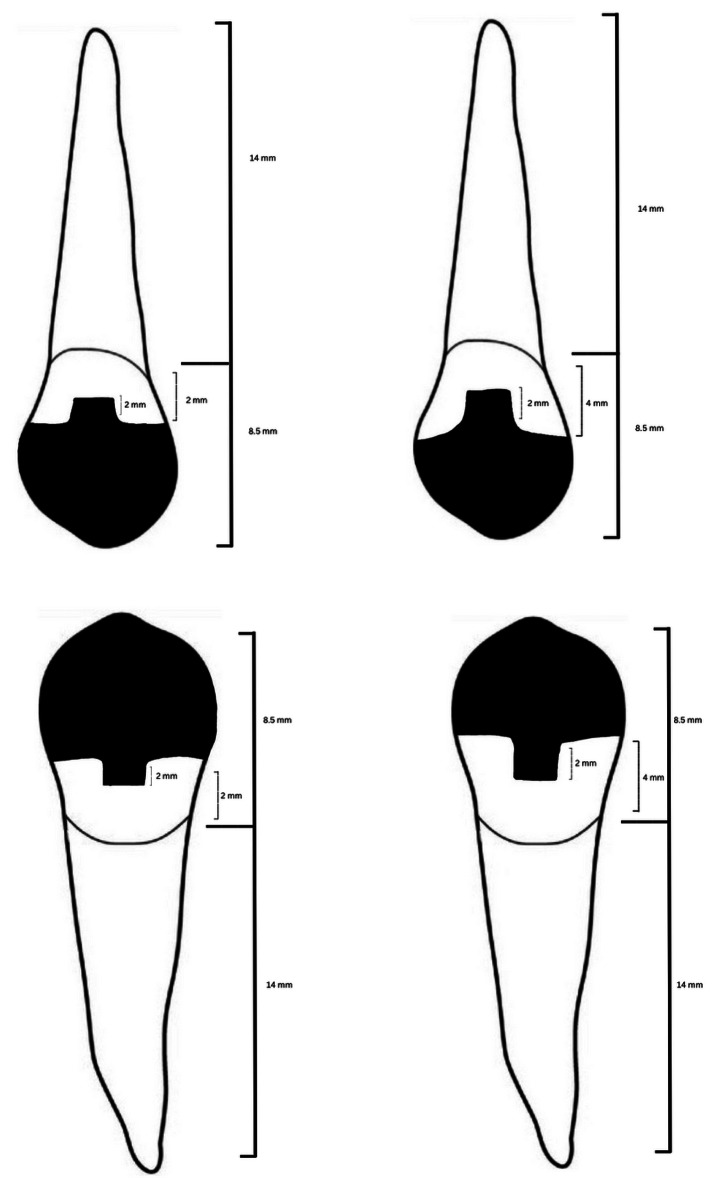
Maxillary and mandibular teeth with different axial height and EC

The 40 samples were randomly distributed into groups comprising 20 each according to premolar type (maxillary or mandibular). Subsequently, extracted tooth samples from each group were divided into two subgroups (*n* = 10) depending on the remaining axial height (2 and 4 mm). Subgroups I and II had axial preparation heights of 2 and 4 mm, respectively (the remaining buccal, lingual, mesial, and distal axial walls). The maxillary or mandibular premolars were sectioned by Komet diamond burs (USA’s, Coarse – Green with Particle grit size: 180 microns) and divided into two groups according to the remaining axial wall height (2 and 4 mm from the CEJ) with an extension of 2 mm inside the pulpal chamber (Fig. 1A&B). Then, all the walls of the pulpal champers were slightly diverged occlusal (Fig. 2) visually.

### Pulp chamber treatment

Pulp treatments were performed as described in the literature [5, 23, 32]. All tooth samples were disinfected with 5 mL of 1% Sodium Hypochlorite, subjected to distilled water irrigation, and dried with paper points, and then the orifices were sealed completely with flowable composite Tetric NFlow (Ivoclar Vivadent, NY, USA. Coronal access was filled with a temporary filling and stocked at 37 °C and 100% humidity for 1 week.

### Zirconia Endocrown constructions and cementation

CAM scanned teeth with different axial heights and margins and then with a desktop scanner (Ceramill Map 400; AmannGirrbach, Herrschaftswiesen, Koblach, Austria), and the data were converted into standard tessellation language (STL) format by using CAD software (3Shape A/S, Holmens Kanal 7, Copenhagen, Denmark) for the design and fabrication of EC restorations separately. According to the CAD machine instruction, the virtual EC with 0.20 μm luting space was designed for each sample. Crowns were fabricated using a five-axis milling machine (Ceramill Matik, Amann Girrbach, Herrschaftswiesen, Koblach, Austria) following the manufacturer’s recommendation [20]. The final endocrowns were almost similar dimensions to the original shape and anatomy of used natural teeth.

For EC cementation, the fitting surfaces of the Ceramill Zolid PS zirconia ECs (Amann Girrbach, North America, Charlotte, USA) were airborne particles abraded with 50 μm aluminium oxide (Polidental Indústria e Comércio, Cotia, SP) at 2.5 bar (5 mm distance) for 3 s, cleaned in an ultrasonic bath of 99% isopropanol for 3 min, and air-dried. The self-etching 3 M Primer 94 (3 M, St. Paul, MN, USA) was mixed, applied on the tooth surface, scrubbed with a micro brush for 15 s, and gently air-dried. After the surface treatments, the ECs were cemented with self-adhesive resin cement (3 M RelyX Unicem 2 Automix, St. Paul, MN, USA) under a constant load seated for 5 min. All the steps of EC cementation were carried out according to the manufacturer’s instructions. Finally, excess cement was removed from each EC and then light polymerized for 60 s using an LED polymerization light (Ultradent Products). The specimens were stored in distilled water at 37 °C for 72 h before the colour test.

### Color measurements

Colour measurements were performed over a grey background for all the samples using a single operator (M. M.) with the help of an Easyshade Vita probe spectrophotometer (VITA Easyshade III, Vita Zahnfabrik, BadSäckingen, Germany). All the samples were measured for the CIE-Lab values to provide the numerical values of the 3D colour measurements. L*, a*, and b* values for all the samples were measured three times, and the average value was considered the ΔE* value. As discussed previously, the values were presented as the means of colour change values and standard deviation [9, 11, 33]. The samples were stained for 2 weeks with coffee, which was changed every 12 h. The device was calibrated after each measurement. The measurements were carried out at the centre of the occlusal surfaces of the crowns after mounting them on white clay material.

The CIELAB colour system, which provides the mathematical values for 3D colour measurements, was used to record the parameters for each premolar ECs [7–11]. The colour readings of the restorations were measured three times by a single operator M.M: at the baseline (L1*, a1*, and b1*) and after 15 days (L2*, a2*, and b2*). The means of the three readings were taken. The mean colour differences between premolars using the following equation:

ΔE*= (L1*- L2*)^2^ +(a1*-a2*)^2^+(b1*- b2*)^2^ × 1/2 where the average colour change values denoted by ΔE*, and L1*, a1*, b1* are the colour coordinates of the EC samples at the baseline, and L2*, a2*, and b2* are the colour coordinates of the EC samples after coffee staining [7, 11].

### Marginal adaptation measurements in micrometres

**Fig. 3:**
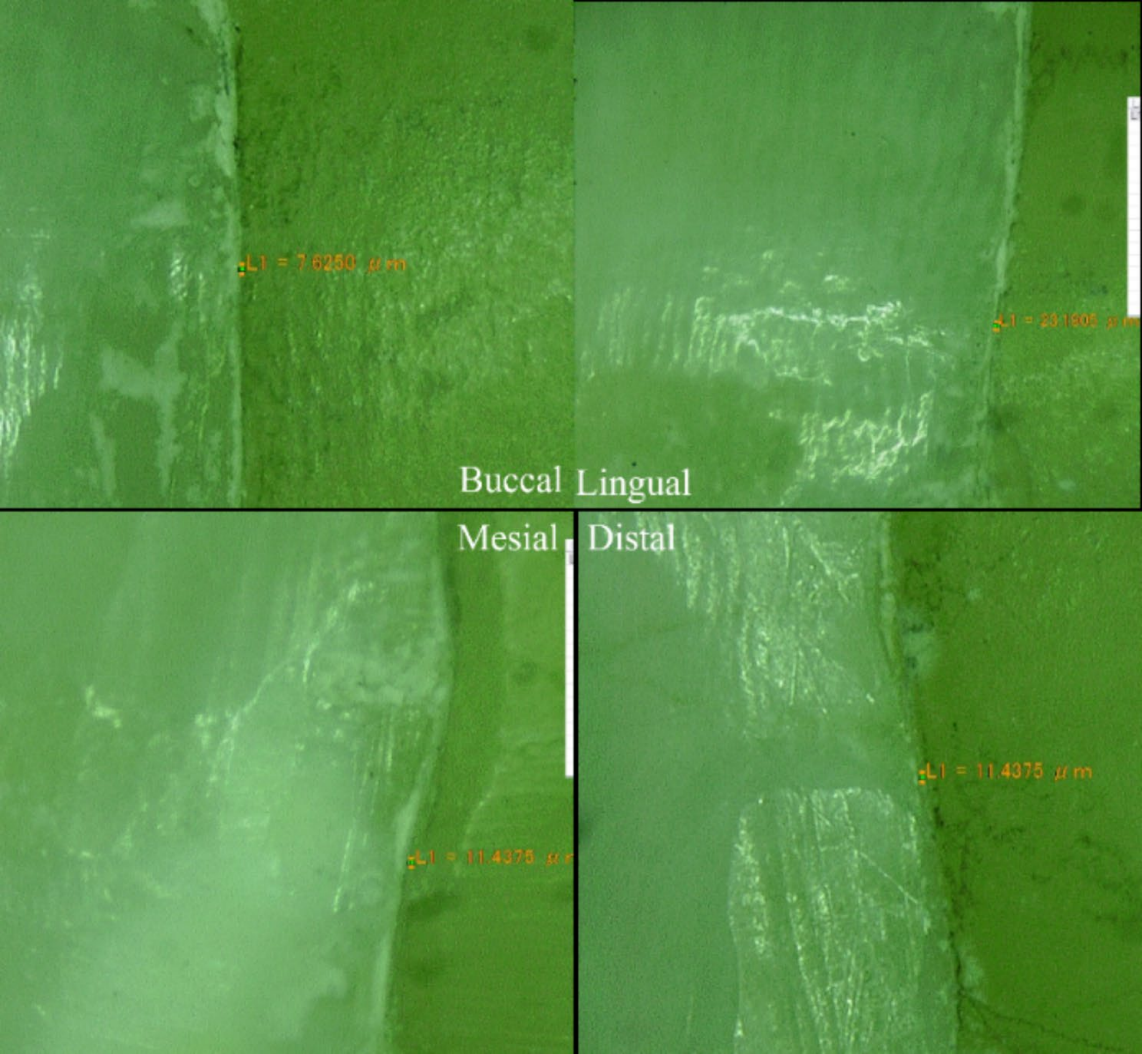
Marginal adaptation from four different sides of the sample

Marginal adaptation was assessed by measuring the vertical distance between the EC margins and the prepared butt joint of teeth. All the samples locked in the locking device were examined at 40× magnification under the lens of a digital microscope (Digital Microscope, KH-7700, Hirox-USA, Inc., USA). The digital camera, light source, liquid-crystal display monitor, computer, and software were all integrated into this microscope. Marginal gap assessments were measured for each shot (four equidistant landmarks along the cervical circumference for each specimen surface: mesial, buccal, distal, and lingual). Each measurement was repeated three times. The images were captured and automatically transferred to digital imaging software, and the lens was calibrated each time. The average values in micrometres were calculated using the measurement software available in the stereomicroscope, which was used to calculate the range values at the measurement points [4, 34], as shown in Fig. 3.

### Fracture force assessments

All the premolar EC samples were subjected to ageing and 5000 thermocycles by a particular machine (Thermocycler, SD Mechatronik, Feldkirchen-Westerham, Germany) in cold (5 °C) and hot water (55 °C) successively. The dwell time was 30 s, and fracture tests were performed [11, 34]. After 24 h, we placed the samples in a vice fixture on a Universal Testing Machine (Instron, Norwood, MA, USA) to direct the static load at a 1 mm/min crosshead speed along the oblique axis with 45 angles of the mounted premolar EC. A thin paper covered the occlusal surface to ensure the uniform distribution of the fracture loaded with a 3 mm diameter hardened stainless steel piston with a 0.5 m radius at a rate of 0.5 mm/min. The highest load at fracture was documented in Newton [20, 29, 30].

### Fracture mode assessment

After the thermocycling period and fracture forces test, we examined all specimens visually under a stereomicroscope (Olympus/DeTrey, Germany) at 10× magnification to assess and record the failure mode and determine the fracture origin. Failure modes were classified into three types [20]: type I failure, ceramic fracture or debonding of the EC; type II failure, ceramic and tooth fracture below the CEJ; and type III failure, splitting fracture.

### Statistical analysis

The data were entered and analyzed using the Statistical Package for Social Sciences for Windows (version 28.0.; Armonk, NY: IBM Corp). The confidence intervals were 95%, and a p-value ≤ 0.05 was considered statistically significant. An unpaired t-test was used to compare mean colour change, marginal adaptation, and fracture forces.

## Results

**Fig. 4:**
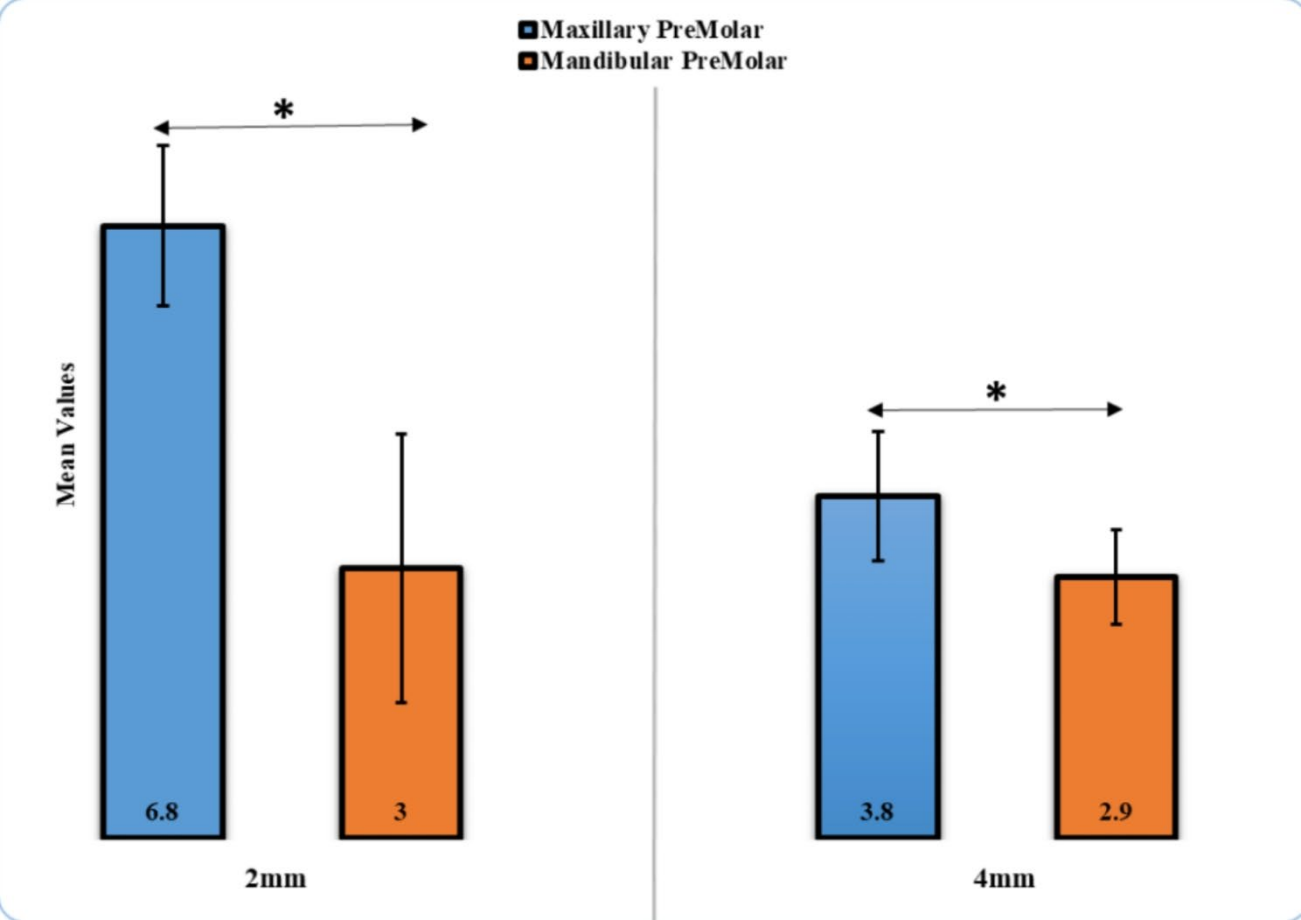
Comparison of Color Change for EC Premolar with 2- and 4-mm height

**Fig. 5:**
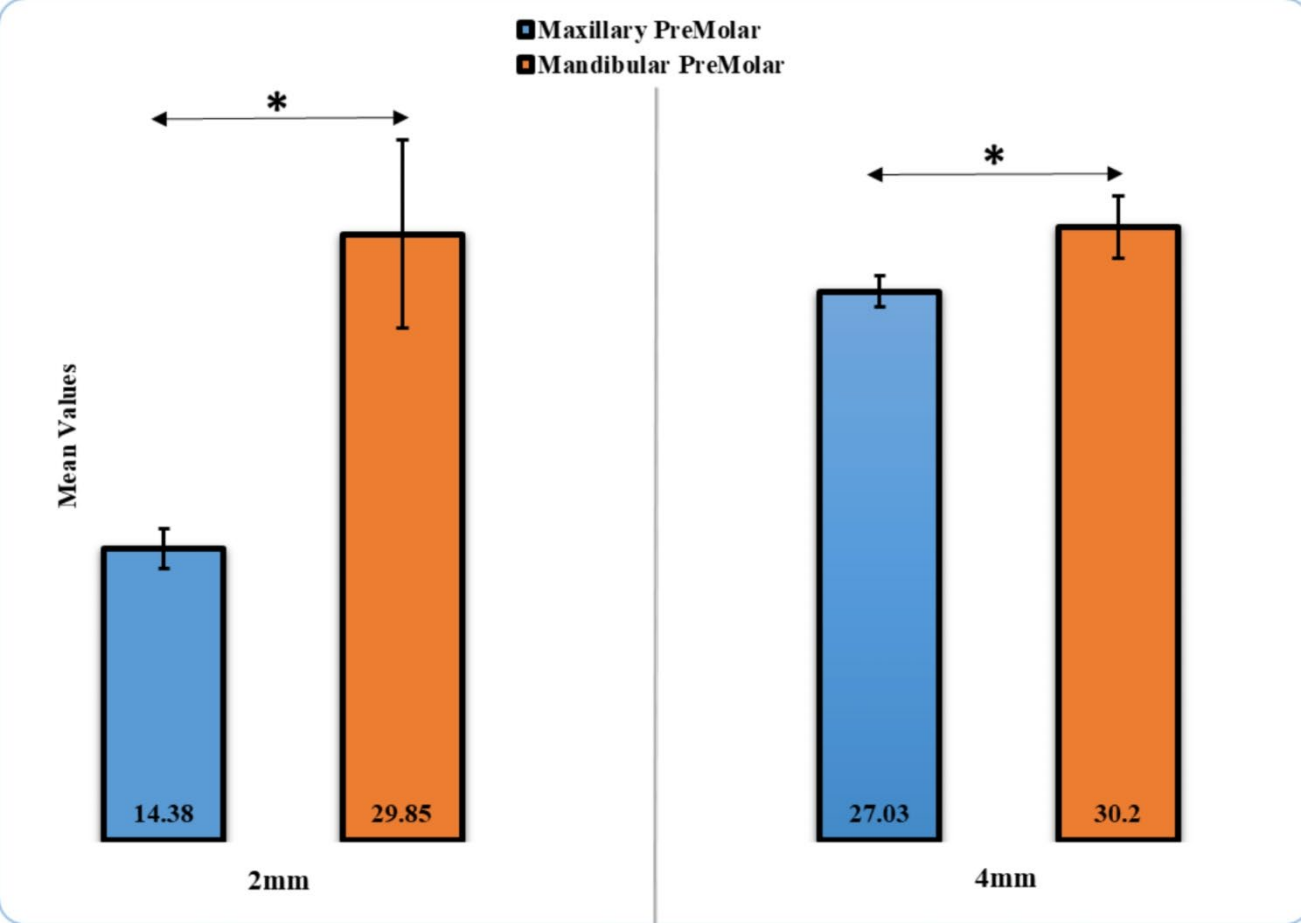
Comparison of Marginal Adaptation in µm for Maxillary and Mandibular EC Premolar at 2 and 4 mm

The mean colour change (ΔE*) values of the maxillary premolar at 2 mm were the highest (6.8 ± 0.89), whereas the lowest ΔE* value was observed in the mandibular premolar at 4 mm (2.9 ± 0.53). The statistically significant difference in ΔE* (*p* < 0.05) was found between maxillary and mandibular premolars at 2 and 4 mm (Table 1; Fig. 4). The marginal adaptation of the mandibular premolar at 4 mm was higher (30.20 ± 1.53 μm), in comparison with that recorded for the maxillary premolar at 2 mm (14.38 ± 0.99 μm). A statistically significant difference (*p* < 0.05) was detected between maxillary and mandibular premolars at 2 and 4 mm (Table 2; Fig. 5).

**Table 1:** Comparison of Color Change (ΔE* units) for Maxillary and Mandibular Premolar EC with 2 and 4 mm

Color change	Mean	Std. deviation	*p*-value
Maxillary premolar, 2 mm	6.8	0.89	0.001*
Mandibular premolar, 2 mm	3.0	1.49	
Maxillary premolar, 4 mm	3.8	0.72	0.02*
Mandibular premolar, 4 mm	2.9	0.53	

*Significant

**Table 2:** Comparison of marginal adaptation in µm for Maxillary and Mandibular Premolar at 2 and 4 mm

Marginal Adaptation	Mean	Std. Deviation	*p*-value
Maxillary Premolar, 2 mm	14.38	0.99	0.01*
Mandibular Premolar, 2 mm	29.85	4.63	
Maxillary Premolar, 4 mm	27.03	0.78	0.01*
Mandibular Premolar, 4 mm	30.20	1.53	

**Fig. 6:**
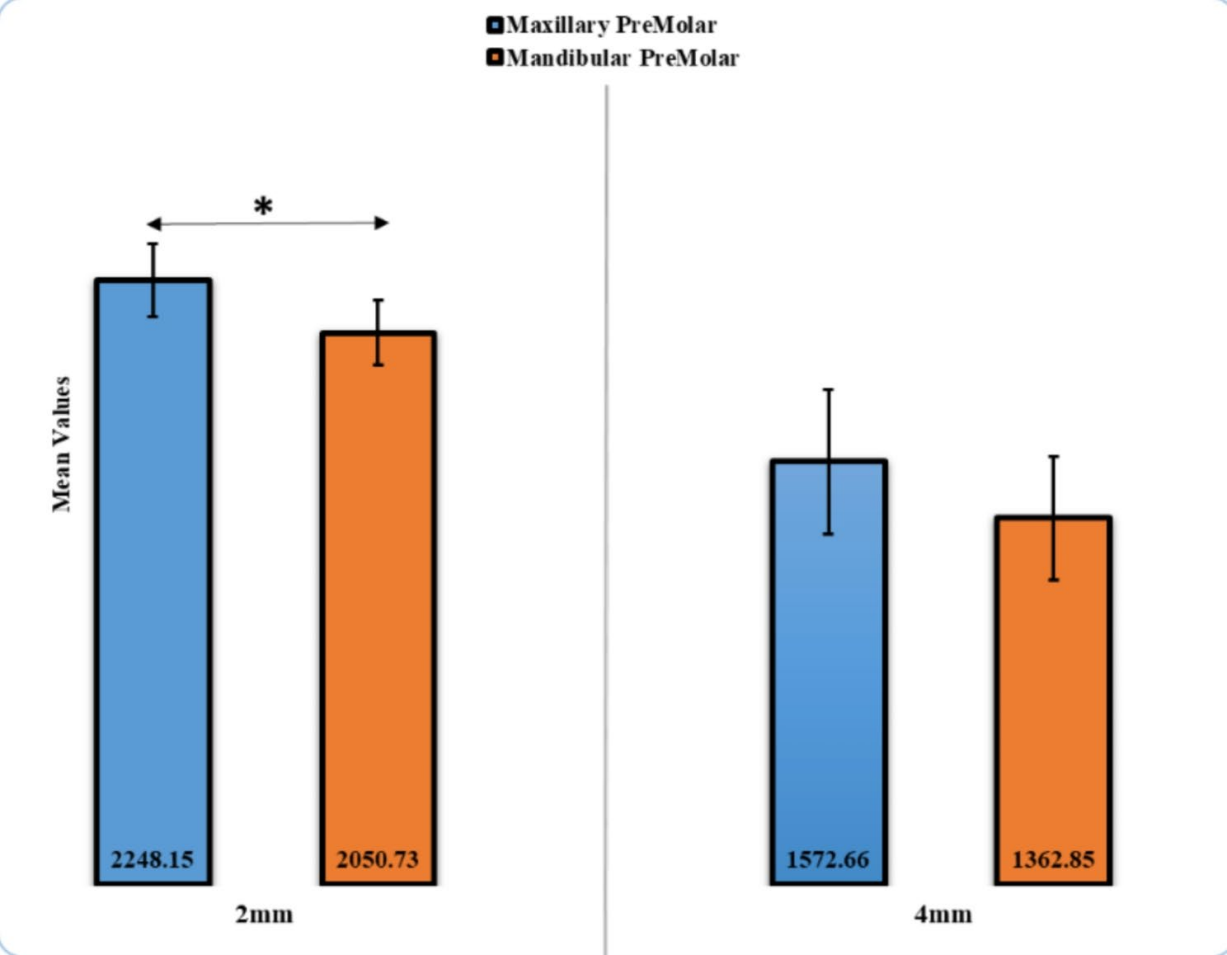
Comparison of Fracture Forces Values for EC Premolar at 2 and 4 mm

**Fig. 7:**
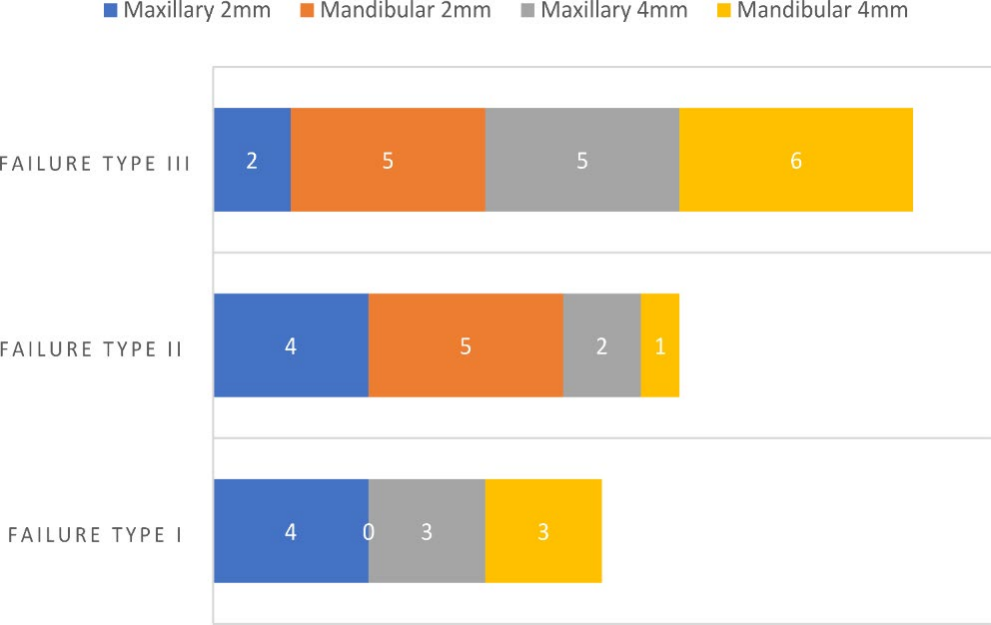
Fracture types in premolars

**Fig. 8:**
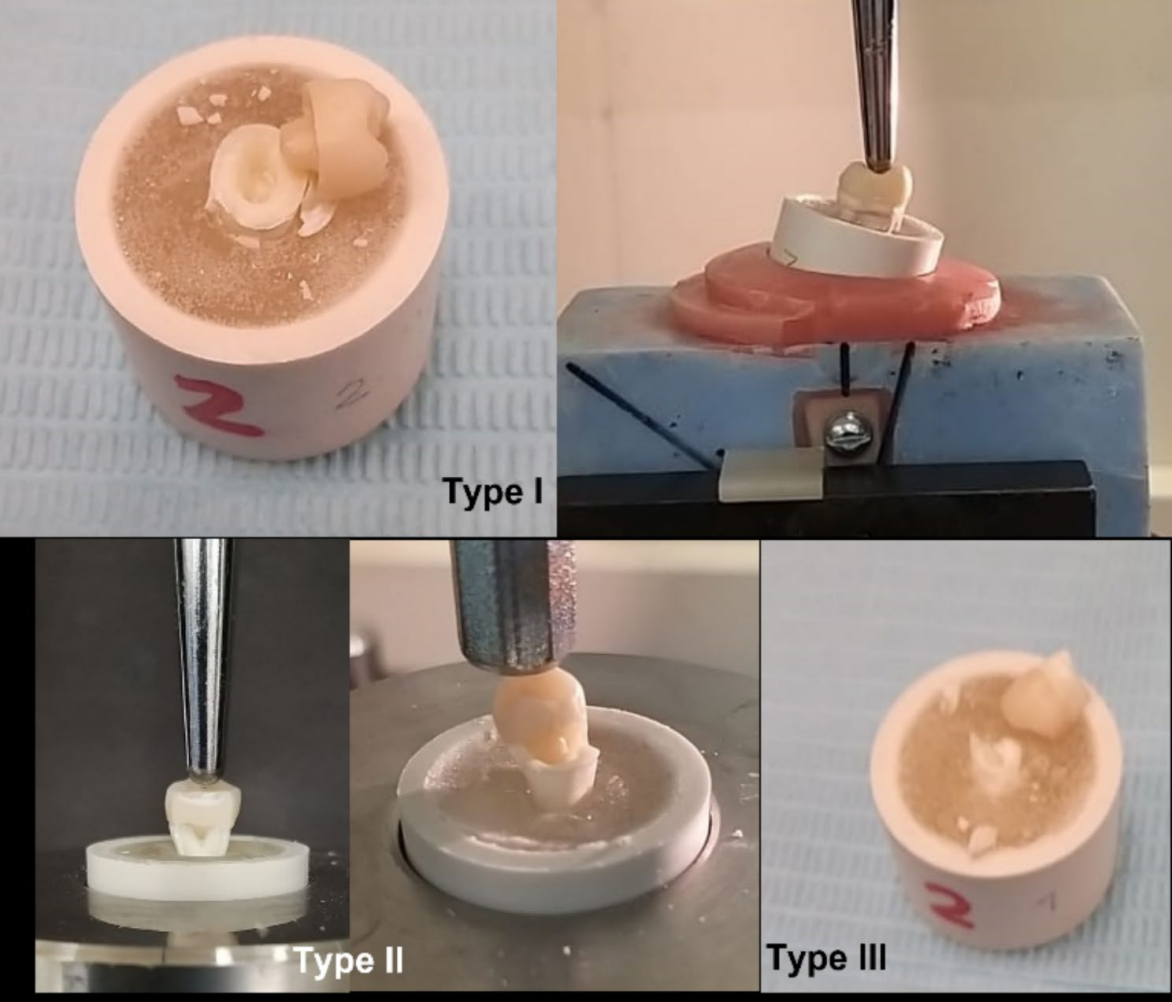
Failure mode type. Type I (ceramic fracture or debonding) and Type II fracture (ceramic and tooth fracture below the CEJ), Type III failure (split fracture)

The maximum fracture force was observed in the maxillary premolar with 2 mm axial height (2248.15 ± 134.74 N), and no statistically significant difference (*p* > 0.05) was found between maxillary and mandibular premolars at 4 mm axial height (Table 3; Fig. 6). Regarding fracture type, almost 60% of Type III failure (split fracture) had the highest type of failure in maxillary and mandibular premolar ECs with different axial wall heights, except maxillary premolar EC with 2 mm axial height, which had a higher fracture type in Types I (ceramic fracture) and Type II fracture (ceramic and tooth fracture below the CEJ; Fig. 7) (Table 4). Digital images are presented in Fig. 8 for each failure mode.

**Table 3:** Comparison of Fracture forces values in N for EC Premolar at 2 and 4 mm

Fracture force	Mean	Std. deviation	*p*-value
Maxillary premolar, 2 mm	2248.15	134.74	0.03*
Mandibular premolar, 2 mm	2050.73	120.42	
Maxillary premolar, 4 mm	1572.66	269.30	0.07
Mandibular premolar, 4 mm	1362.85	230.70	

**Table 4:** Fracture types in endocrown premolars

Premolar	Failure type I	Failure type II	Failure type III
Maxillary, 2 mm	4	4	2
Mandibular, 2 mm	0	5	5
Maxillary, 4 mm	3	2	5
Mandibular, 4 mm	3	1	6

## Discussion

The present study evaluated the effect of different preparation designs and tooth types (maxillary and mandibular premolars) on colour change, marginal adaptation, fracture strength, and Ceramill Zolid PS zirconia EC failure type. The oral environment was simulated through thermomechanical ageing. The different preparation designs of premolar EC in the present study were 2- and 4-mm pulpal extension because a flat EC with no pulpal extension results in poor outcomes [35].

The recorded ΔE* after coffee immersion was between 2.9 and 6.8, which is acceptable and slightly higher than the adequate ΔE* values (1.8–4.2 units) [11]. Significant differences were found among different EC designs; the null hypothesis that the values of mean colour change, marginal adaptation, fracture forces, and type percentages among EC restorations will not vary was rejected.

A spectrophotometer was utilized for ΔE* measurement because of its precision, numerical expression of colour, and lack of subjective bias [36, 37]. Due to the different parameters used to evaluate ΔE* independently, many possible colours in three-dimensional real number space can be measured [10, 36, 37]. The chemical structure, matrix, matrix/load interphase variation, and physical chemical reactions are intrinsic factors that determine the colour of CAD/CAM ceramic material [38]. The ECs of the current study were fabricated by CAD/CAM technology using zirconia, and the colour was assessed using a spectrophotometer. A significant difference was recorded in ΔE* between the different designs; this could be related to the thickness of restorations at the area of measuring the colour (occlusal surfaces with the presence of an anatomical groove of the ECs).

The ΔE* values recorded in the current study agree with those reported by Kang et al. [14] because most samples in the current study were as thick as or thicker than their samples. The ΔE* values recorded by Aldosari et al. [7] and Alghazali et al. [9] after coffee immersion on zirconia CAD/CAM materials were lower than those obtained in the current study. The present study results were consistent with the coffee immersion study of Haraluer et al. [39], who showed that the ΔE values related to ageing were high in zirconia. In agreement with the ΔE values by Al Ahmari et al. [10], the ΔE values in the current study were between 3.0 units for different EC designs. These values can be attributed to the thickness and composition of the CAD/CAM ceramic materials used in all studies after immersion in coffee solutions. EC’s recorded ΔE values were marginally higher than the acceptable clinical value (1.8–4.2) [11]. This result was further clarified by Alrabeah et al. [12]. Agreeable mean values may have occurred because the uneven surface of the unglazed pressed ceramic promoted water infiltration and subsequent silica network disintegration, reduced crystallinity, and increased absorption of colouring pigments.

The marginal adaptation of extra-coronal restoration considerably influences the long-lasting dental restoration service. The poor marginal adaptation (high marginal gap) can result in the exposure of cement to oral fluids, leakage, plaque accumulation, secondary caries, periodontal inflammation, and the complete failure of prosthodontic treatment [40–42].

The mean of marginal adaptation was 14.38 μm for 2 mm maxillary premolar EC and 29.85 μm for the 2 mm mandibular premolar EC. The mean of marginal adaptation was 27.03 μm for 4 mm maxillary premolar EC and 30.20 μm for 4 mm mandibular premolar EC. The second null hypothesis in the present study was rejected because of the differences between the mean of marginal adaptation for 2 mm maxillary premolar EC and 2 mm mandibular premolar molar EC (*p* = 0.01) and between the mean of marginal adaptation for 4 mm maxillary premolar EC and 4 mm mandibular premolar EC (*p* = 0.01). The significant difference may be associated with the thickness of the remaining cemented surfaces of the endocrown. Also, the marginal adaptation is the overall of the four surfaces.

In the present study, the marginal adaptation value of the 4 mm maxillary premolar EC was similar to the findings of Soliman et al. [4], who reported that the marginal adaptation values of the 3 mm maxillary premolar LDGC EC reinforced by machinable zirconia was 29.54 μm. Taha et al. [5] found that the marginal adaptation value (73.1 μm) after the cementation of 6 mm mandibular premolar EC with lithium silicate reinforced by machinable zirconia was higher than that obtained in the present study. A review published in 2023 showed that the average marginal adaptation values of zirconia EC were 62.34 and 73.17 μm for CAD/CAM and pressable zirconia materials, respectively [43]. These values were slightly higher than those recorded for CAD/CAM EC in this study, regardless of tooth position or the presence or absence of an EC extension inside the pulp.

Studying the mechanical properties of zirconia CAD/CAM materials is essential to evaluating their clinical behaviour. The mean fracture forces in the present study were 2248.15 N for 2 mm maxillary premolar EC and 2050.73 N for 2 mm mandibular premolar EC. The mean of fracture forces was 1572.66 N for 4 mm maxillary premolar EC and 1362.85 N for 4 mm mandibular premolar EC. The third null hypothesis for the present study was partially rejected because the difference between the mean of fracture forces for 2 mm maxillary premolar EC and 2 mm mandibular premolar EC was significant (*p* = 0.03). No significant difference was found between the mean of fracture forces for 4 mm maxillary premolar EC and 4 mm mandibular premolar EC (*p* = 0.07). These differences can be related to the extension of the EC design inside a pulp chamber. Slight differences between the size of the bonded surface area of the examined teeth with its ECs, which may cause such differences.

The values of fracture forces in the present study were higher than those reported by many earlier studies [20–22]. Hassouneh et al. [22] found that the fracture force for 4 mm mandibular premolar pulpal extension zirconia EC was 460 N. Ahmed et al. [21] reported that the fracture force for 4 mm maxillary premolar pupal extension with no ferrule zirconia EC was 1391 N. Furthermore, Demachkia et al. [20] found that the fracture force for 2 mm maxillary premolar pulpal extension and four axial wall zirconia EC was 1486.7 N. Al Fodeh et al. [44] reported a 1334 ± 332 N value by testing maxillary premolar zirconia ECs. All these values are equal to or lower than the values recorded in the current study, which ranged from 1362.85 N to 2248.15 N. Variations in the fracture forces of zirconia EC among the studies can be explained by heterogeneity in terms of fracture strength assessment protocol, manufacturer, restorative material, sample size, and remaining tooth structure [45].

Failure type indicates restoration’s clinical performance and durability in the oral cavity before fracture [10]. The null hypothesis was rejected because combinations of failure types with different percentages were found in different designs. Most failure types in the 2- and 4-mm maxillary and mandibular premolar ECs were catastrophic (types III and II). This finding agrees with previously reported results [20–22, 44, 46]. The high percentage of catastrophic failure of zirconia EC can be explained by the higher modulus of elasticity of monolithic zirconia ceramics compared with dentin.

### Limitation and future scope of the study

Since this study included only two axial wall height designs and one pulpal extension length, this can be considered a limitation. Also, only single material was tested. These designs can be highly recommended for clinical use in premolars after RCT as an alternative restoration to conventional full-coverage crowns. Further studies can be performed with different designs, such as pulpal extension, axial wall height, recent types of materials, and exposing the samples for a long thermocycling period. Also, clinical studies are highly recommended to include such oral environment and conditions involved in the study, which can be considered a limitation in this study, such as pH levels, microbial presence, and other dynamic factors.

## Conclusions

Within the limitations of this laboratory study in investigating the optical and mechanical properties of zirconia ECs. The following conclusions were obtained:

1. Most of the ΔE* values after coffee staining were within clinically acceptable values in the mandibular EC samples and were slightly higher than those in the maxillary premolar ECs (1.8–4.2).
2. The marginal adaptation of the maxillary and mandibular premolars ranged from 14.38 μm to 30.20 μm and was considered clinically acceptable (0.00–120 μm).
3. The mean fracture forces were high in the maxillary and mandibular ECs with a 2 mm extension in the pulp chamber.
4. Failure type III (split fracture) had the highest frequency in maxillary and mandibular premolar ECs with different axial wall heights, except maxillary premolar EC with 2 mm axial height, which showed a high degree of Type I (ceramic fracture) and Type II failure (ceramic and tooth fracture below the CEJ).

## Data Availability

All data supporting the findings of this study are available from the corresponding author upon reasonable request.
